# Structure and properties of virions and virus-like particles derived from the coat protein of Alternanthera mosaic virus

**DOI:** 10.1371/journal.pone.0183824

**Published:** 2017-08-24

**Authors:** Ekaterina K. Donchenko, Evgeniya V. Pechnikova, Maryia Yu. Mishyna, Tatiana I. Manukhova, Olga S. Sokolova, Nikolai A. Nikitin, Joseph G. Atabekov, Olga V. Karpova

**Affiliations:** 1 Faculty of Biology, Lomonosov Moscow State University, Moscow, Russia; 2 Laboratory of Electron Microscopy, V.A. Shoubnikov Institute of Crystallography of Russian Academy of Sciences, Moscow, Russia; 3 Nano-, Bio-, Information, Cognitive, Socio-Humanistic (NBICS) Science and Technology Center, National Research Centre "Kurchatov Institute", Moscow, Russia; 4 Department of Food Science, Tel Hai College, Upper Galilee, Israel; University of California, Riverside, UNITED STATES

## Abstract

Plant viruses and their virus-like particles (VLPs) have a lot of advantages for biotechnological applications including complete safety for humans. Alternanthera mosaic virus (AltMV) is a potentially promising object for design of novel materials. The 3D structures of AltMV virions and its VLPs were obtained by single particle EM at ~13Å resolution. The comparison of the reconstructions and a trypsin treatment revealed that AltMV CPs possesses a different fold in the presence (virions) and absence of viral RNA (VLPs). For the first time, the structure of morphologically similar virions and virus-like particles based on the coat protein of a helical filamentous plant virus is shown to be different. Despite this, both AltMV virions and VLPs are stable in a wide range of conditions. To provide a large amount of AltMV for biotechnology usage the isolation procedure was modified.

## Introduction

Alternanthera mosaic virus (AltMV) is a plant virus belonging to the genus *Potexvirus*, family *Alphaflexiviridae*. Flexible filamentous virions of AltMV have helical type of symmetry; they are ~570 nm long and ~ 13 nm in diameter. Virus contains a positive single-strand RNA, 6606 nt long. AltMV was for the first time isolated and described in Australia in 1999 [1], but later AltMV isolates were also reported in Europe [2], the USA [3–7], Brazil [8] and Asia [9]. A new strain of AltMV–AltMV-MU (Moscow University) (accession number FJ822136 in the GenBank) was characterized in our laboratory in 2011 [10]. AltMV-MU RNA contains a cap at its 5' end and a poly(A) sequence at its 3' end, and encodes five proteins: a 174 kDa viral replicase, three movement proteins (the products of “Triple Gene Block” with molecular weights of 26 kDa, 12 kDa and 7 kDa) and a 22 kDa coat protein (CP) [10]. According to its serological properties and on the basis of the nucleotide and amino acid sequences similarity, AltMV is the closest relative of the papaya mosaic virus (PapMV) [1,5].

In contrast to rich information on the rigid rod plant viruses, structural information for flexible plant viruses has been lacking for long time. Now, the high-resolution structures are available for PapMV [11], bamboo mosaic virus [12] and pepino mosaic virus [13]. The low-resolution structures for a number of other Potexviruses exist, but the 3D structure of AltMV virion has been lacking until now.

Similarly to PapMV CP [14], AltMV CP polymerizes to form extended RNA-free virus-like particles (VLPs) *in vitro* at pH 4.0 and low ionic strength [15]. However, the PapMV CP does not form VLPs at pH 8.0 in the absence of RNA, while the AltMV CP under these conditions forms VLPs morphologically close to native AltMV virions [15].

Recently, plant viruses and VLPs, derived from them, have been used extensively in biotechnology including the development of medical products (vaccines) [16–19]. Plant viruses are absolutely safe for humans, due to the low risk of cross-contamination with mammalian pathogens [20]. Usage of plant viruses as a basis for medical nanotechnologies reduces many of the risks associated with other biological materials, being emplemented in vaccine, as well as the use of noninfective VLPs results in low risk to the environment and human beings. These lower risks allow for easier handling, transporting and processing of viral nanoparticles, making plant virus-based particles particularly attractive platforms for a range of nanobiotechnological applications [19]. Virions and VLPs of PapMV (closely related to AltMV) were shown to have high immunostimulating properties [21–23]. Thereby, the study of the structure and stability of AltMV virions and VLPs can be a promising and tremendously important direction of research.

This work is devoted to investigation of the possibility of obtaining AltMV VLPs under different conditions and to a detailed characterization of AltMV virions and VLPs properties in terms of their biotechnological potential. In addition, the method of AltMV isolation and purification was modified, markedly increasing the yield of the purified virus.

## Materials and methods

### Virus purification

The initial infected material was received from the German Collection of Microorganisms and Cell Cultures, Braunschweig as a strain of PapMV. We propagated this virus and defined it as a new strain of AltMV–AltMV-MU (Moscow University) (accession number FJ822136 in the GenBank) [10].

The virus was maintained in either *Nicotiana benthamiana* or *Portulaca grandiflora*. Plants were provided by the greenhouse of Faculty of Biology, Lomonosov Moscow State University. Plants were inoculated using sap extracts (c. 1:5–1:10, w:v of 0.01 M Tris-HCl, pH 7.5, with Celite added as an abrasive) from plant tissue of infected *P*. *grandiflora*. 30 days after infection, the plant biomass was frozen at -20°C. For virus purification, plants were homogenized in 0.3 M glycine-KOH, 1% Na2SO3, pH 7.5 (3 ml buffer per 1 g of leaves) in a blender. All steps of virus preparations were carried out at 4°C and with chilled buffers. The plant sap was then filtered through a cheesecloth, and the filtrate centrifuged at 12,000 rpm for 30 min (Beckman J2-21 Centrifuge, JA-14 rotor). The supernatant was collected and clarified with Triton X-100 added to 1% (v/v) and incubated for 20 min. Polyethylene glycol (PEG; MW 6000) and NaCl were added to the solution to 5% and to 2% (w/v), respectively. After dissolving the components, the solution was kept overnight at +4°C. The PEG precipitate was collected by centrifugation at 10,000 rpm for 20 min (Beckman J2-21 Centrifuge, JA-14 rotor). The sediment was collected and retained at +4°C. The PEG (Mr 6000) was added to the supernatant to a final concentration of 8% (taking into account the one added earlier). After dissolving the components, the solution was kept overnight at +4°C. The PEG precipitate was collected by centrifugation at 10,000 rpm for 20 min (Beckman J2-21 Centrifuge, JA-14 rotor) and combined with the virus precipitate obtained on the previous day.

Extraction of the obtained precipitate was carried out two to three times for 2–6 hours by gentle shaking (until the precipitate was completely dissolved) with a buffer containing 0.05 M Tris-HC1, 0.01 M EDTA, pH 8.0. The resulting extract was centrifugated at 10,000 rpm for 20 min (Beckman J2-21 Centrifuge, JA-14 rotor). The virus suspension was then centrifuged at 110 000 g for 3 h (Hitachi Koki himac CP100WX Ultracentrifuge, P50AT2 or P70AT2 rotor). The pellets were suspended overnight at 4°C in 0.05 M Tris-HC1, 0.01 M EDTA, pH 8.0 and clarified by centrifugation at 10,000 g for 20 min. Then 8 ml of the virus suspension per 10 ml S303922A - 10PC BOTTLE (B) tube was underlaid with 2 ml of 30% sucrose (w/v, in the extraction buffer). Samples were centrifugated at 110,000 g for 4 h (Hitachi Koki himac CP100WX Ultracentrifuge, P70AT2 rotor). The pellets were resuspended in 0.025 M Tris-HCl buffer, pH 8.0; 0.01 M Tris-HCl, pH 7.5 or water. The resulting virus solution was centrifugated at 10,000 g for 20 min. Virion concentration was estimated as described [1,10]. The viral preparation was stored at 4°C or frozen at -20°C for long-term storage.

### Coat protein isolation

The method of salt deproteinization with 2M LiCl as described previously [10,24].

### Stability analysis of AltMV virions and virus-like particles

The AltMV virions and virus-like particles were incubated at room temperature for 1 hour under different conditions (distilled water; 0.15 M NaCl; 0.01 M Tris-HCl, pH 7.5; 0.01 M Tris-HCl, 0.15 M NaCl, pH 7.5; mouse serum). Results were analyzed by electron microscopy.

### Electron microscopy

The samples were prepared as described by Nikitin et al. [25] and examined either in a transmission electron microscope JEOL JEM-1400 TEM (JEOL, Japan) operating at 80 kV, or JEOL JEM-2100 (JEOL, Japan) operating at 200 kV. Images were recorded on an Olympus Quemesa digital camera under the control of iTEM software (Olympus Soft Imaging Solutions GmbH, Germany). The length and width of viral and virus-like particles were measured manually from digital prints using scientific image manipulation software ImageJ (National Institutes of Health, USA). To obtain the 3D reconstructions, the images were captured with an Ultrascan 1000XP 4x4 pixel CCD camera (Gatan) at x40,000 magnification with an underfocus of 1.5–2.8 um.

### Cryoelectron microscopy

The AltMV samples were diluted to a final concentration of 1.5mg/ml in 25mM Tris-HCl, pH 7.5. 3.5 μl of the AltMV was applied to glow-discharged Quantifoil 1.2/1.3 grid (Quantifoil Micro Tools GmbH, Germany). The grids were then blotted and frozen in liquid ethane using Vitrobot (Termo Fisher Scientific, USA). Low dose images (20–25 e^−^/Å^2^) were collected on a Tecnai G12 Spirit Twin electron microscope (Termo Fisher Scientific, USA) operated at 120 kV, using a 4x4 Eagle CCD camera (Termo Fisher Scientific, USA). A defocus range between 0.8 and 3.5 μm underfocus was used.

### Image processing

Image processing was carried out using the SPRING software [26]. Briefly, the images of the viruses and VLPs were cut out into 100x100 pixel square segments with an overlap of 360 Å in the EMAN *helixboxer* program [27] and appended in a single stack. The CTF was determined for each micrograph using CTFFIND3 [28] incorporated into the SPRING program. Thereby 1180 single frames were collected for AltMV VLPs, and 230 for AltMV virions. Then, all the AltMV virion particles were classified into 10 classes with ~22 particles per class. AltMV VLPs were classified into 50 classes with ~22 particles per class. For all class sums, the Fourier transform was carried out.

To determine the parameters of the helix, three-dimensional reconstructions with low resolution were constructed at various parameters of the helix angle (from 34 to 44 Å in 0.1 Å steps) and the number of subunits (from 8 to 10 Å in increments of 0.01 Å) per rotation of the helix. As a result, 50 reconstructions were generated. The pitch of the helix was estimated using 50 best reconstructions based on the average cross-correlation index and amounted to 35.7 Å. The determination of the number of subunits per helix is based on an analysis of the amplitude of the correlation between the class-average and the re-projections of the 3D models. The subsequent improvement of the reconstruction was carried out in 50 iterations, until the resolution of the electron density map did not cease to improve.

The resolution of the final 3D reconstructions was estimated by the Fourier shell correlation (FSC) using a 0.143 criterion. This was done by splitting the final average and symmetry related views into even and odd numbers. The reconstructions from the two halves of the data were used in the FSC calculation.

To generate unbiased difference maps, the 3D reconstructions of AltMV virions and VLPs were aligned against their symmetry axis using Chimera 1.9 [29] before subtraction using the command vop substract. To visualize differences between the AltMV virions and VLPs the obtained difference map was fitted into the 3D reconstruction of VLP in Chimera 1.9.

### Trypsin test

The trypsin (Promega, 10 ng per 1 mg of virions, VLPs or CP) was added to the samples, and they were incubated in 100 mM Tris-HCl, pH 8.0 for one hour at 37°C as described in [30].

## Results and discussion

### Optimization of the AltMV purification protocol

For the successful use of plant viruses in biotechnologies, it is necessary to provide the effective accumulation, isolation and purification of the viral particles. The high yield of virus per unit of plant biomass is important to the economic expediency of plant viruses’ usage. Undoubtedly, among plant viruses actively used in biotechnology, the Tobacco mosaic virus remains the "champion" in accumulation in plants: its yield reaches up to 10 g / 1 kg of green plant biomass [31].

The first stage in this work was the optimization of the AltMV purification protocol to produce large quantities of virus. Earlier, AltMV was isolated from *Chenopodium amaranticolor* [1], *Nicotiana benthamiana* [5] and *Portulaca grandiflora* [10,15]. In our present work, to increase the AltMV yield, the previous method of virus isolation and purification has been improved and adapted for two plants: *P*. *grandiflora* and *N*. *benthamiana*. Geering and Thomas [1] propagated AltMV in *C*. *amaranticolor*, yielding a 23.4 mg of virus per 100 g of infected leaves. This is a quite high yield, but the host plant used for this cannot be considered optimal for the accumulation of AltMV. *C*. *amaranticolor* is the host for a wide variety of plant viruses, including the representatives of the *Potexvirus* genus that can lead to contamination of the AltMV with other viruses [32]. Therefore, we decided to use plants of portulaca and tobacco, since *P*. *grandiflora* is hardly susceptible to infection with other viruses [33], and *N*. *benthamiana* is a classical laboratory plant very convenient for work. We conducted a passage of infectious material through *P*. *grandiflora* for obtaining a pure preparation of AltMV to ensure that no coinfection occurs.

AltMV was purified following a procedure initially developed for another potexvirus, Potato virus X, with some modifications [10,15]. Geering and Thomas [1] used the isolation procedure described by Bancroft et al. [34]. Hammond et al. [5] isolated AltMV according to a protocol initially developed for potexviruses, as adapted for purification of numerous potyviruses.

Based on the method described in Mukhamedzhanova et al. [15] and Ivanov et al. [10], we made the following changes to it (see Materials and Methods for details): we introduced one more stage of virus precipitation by PEG (in total, two steps: addition PEG to 5% and addition PEG to 8%, precipitation overnight), increased the number and duration of virus extraction after PEG precipitation (2–3 times for 2–6 hours), increased the duration of high-speed centrifugation. Thus, a homogeneous preparation of high purity AltMV was obtained, and the virus yield was essentially increased (Table 1).

**Table 1:** Yield of AltMV isolated from various plants.

Plant	Yield (mg of a virus/100 g of green plant biomass)	Authors
***Portulaca grandiflora***	3,4	Mukhamedzhanova *et al*., 2011 [15]
	20,0	In the current work
***Nicotiana benthamiana***	8,6–12,4	Hammond *et al*., 2006 [5]
	57,3	In the current work

### Structure and stability of AltMV virions

**Fig 1:**
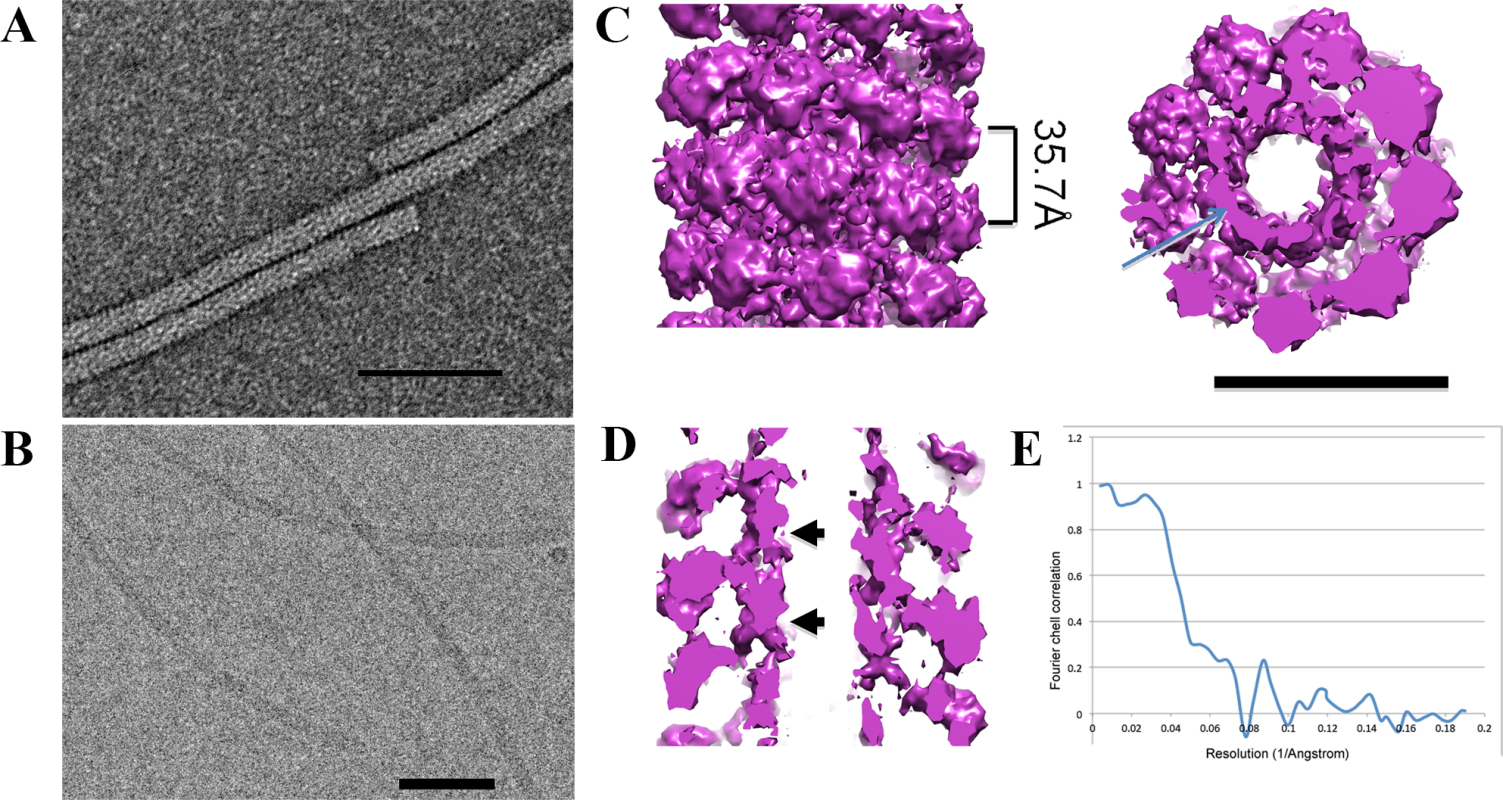
EM analisys of AltMV virions. **A.** Transmission electron microscopy (TEM) of AltMV virions. Samples were stained with 2% uranyl acetate. Bar– 100 nm. **B.** Cryo-electron microscopy of AltMV virions. Bar– 100 nm. **C. 3D reconstruction: on the left–**surface of AltMV virion, **on the right–**horizontal slice of AltMV virion. Bar– 10 nm. **D.** Vertical slice of AltMV virion, possible RNA location is marked with an arrow. **E.** FSC curve.

Images of AltMV viral particles were obtained using electron microscopy (EM), both in negative stain (Fig 1A) and in cryo-conditions (Fig 1B). In both conditions, the viral particles have a somewhat similar diameter of ~135Å (S1A Fig). The helical parameters were determined using the SPRING program [26]: a pitch ~ 35.7Å with ~ 8.75 subunits per turn (Fig 1C).

Using 230 viral segments cut from raw EM images (S1A Fig) the 3D map of the AltMV virion was built using SPRING [26]. The refined map has a resolution of 13Å (Fig 1E) according to Fourier shell correlation (FSC). There is a central channel inside the particle with a ~20Å diameter (Fig 1C) and a density at a ~60Å diameter, apparently resembling a viral RNA (Fig 1C). On the sagittal section (Fig 1D) of the virus reconstruction, it is clearly visible that this density is positioned between subunits in contact with CPs, similar to other plant viruses [35]. Notably, helical parameters of AltMV determined here were similar to those of other Potexviruses with known structure: pepino mosaic virus has a pitch of ~34.6 Å and ~8.7 subunits per turn [13]; bamboo mosaic virus has a pitch of ~ 35 Å with ~ 8.8 subunits per turn [12]. This can be explained by high homology of CPs of these viruses [36]. Moreover, the overall 3D structure of the AltMV virus particle (Fig 1C) is similar in morphology to the known structures of Potexviruses [12,13].

The stability of viral particles in different conditions, especially in conditions close to physiological, is an important factor for their successful use in biotechnology. Analysis by electron microscopy showed that the AltMV virions retain their overall morphology and are stable after incubating them for 1 hour in distilled water (S2A Fig), in 0.15 M NaCl (S2B Fig), and 0.01 M Tris-HCl, 0.15 M NaCl, pH 7.5 (S2C Fig). Moreover, viral particles do not break down when they are incubated in mouse serum (S2D Fig).

### Structure and stability of AltMV VLPs

As it was shown previously, AltMV CP is able to polymerize *in vitro* in the absence of RNA with the formation of VLPs not only at acidic pH 4.0, but also at pH 8.0, which significantly distinguishes it from PapMV CP [14,15]. In the present study, we obtained AltMV VLPs at physiological pH 7.5 that possess a similar structure to the native AltMV virions (S3A and S3C Fig).

**Fig 2:**
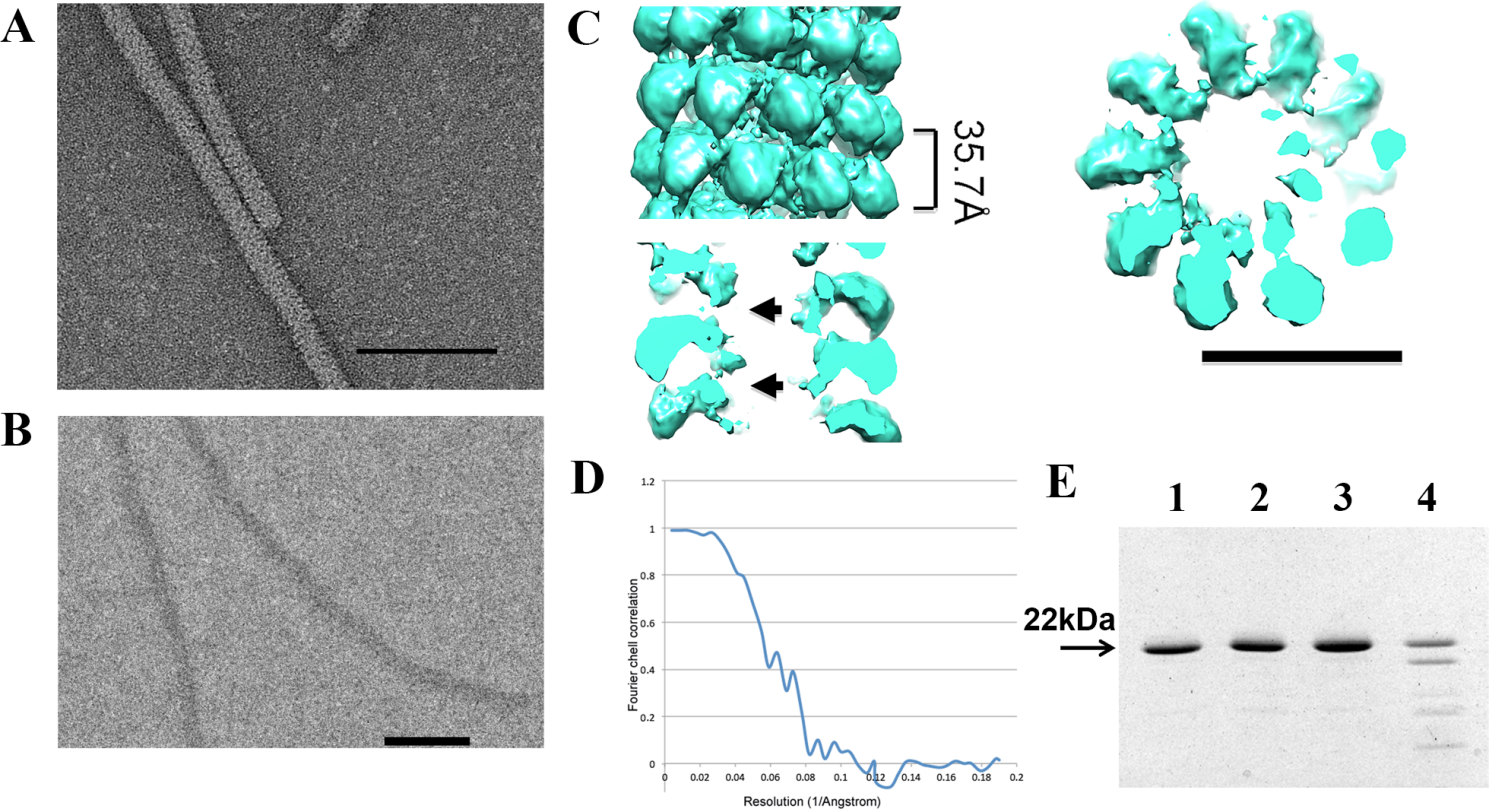
EM analysis of AltMV virus-like particles (VLPs). **А.** TEM of AltMV VLPs. Samples were stained with 2% uranyl acetate. Bar– 100 nm. **B.** Cryo-electron microscopy of AltMV VLPs. **C. 3D reconstruction: on the left–**surface of AltMV VLP, **on the right–**horizontal slice of AltMV VLP, **below**–vertical slice of AltMV VLP. Arrows are pointing to the absence of the density, associated to the viral RNA in Fig 1. **D.** FSC curve. **E.** Structure analysis by trypsin treatment. **1 –**AltMV virions, **2** –AltMV virions treated by trypsin, **3** –AltMV VLPs, **4** –AltMV VLPs treated by trypsin. All samples contained 2 μg of material. Analysis in 8–20% SDS-PAGE, gel was stained with Coomassie G-250.

**Fig 3:**
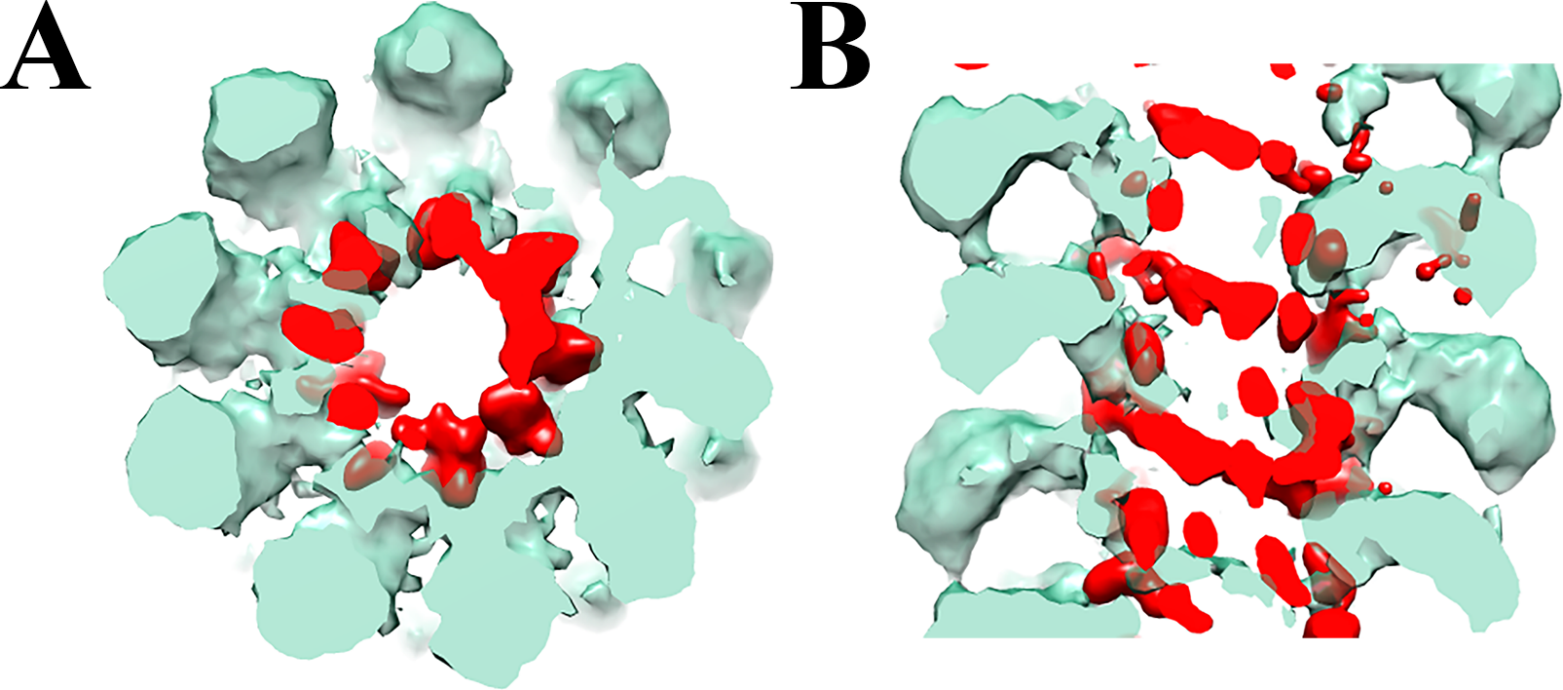
Difference map (red) between aligned to each other AltMV VLP and virion, superimposed onto a 3D structure of a VLP (transparent green). **A.** Sagittal section. **B.** Cross section.

Using single particle EM, we studied VLPs both in negative stain (Fig 2A) and in cryo-conditions (Fig 2B). Again, the VLP particles in both conditions have a similar diameter ~152Å, which is larger comparing to the wild type AltMV virion (S1B Fig). Using SPRING protocol we have estimated the number of subunits per turn. The refined map of the VLP particle (Fig 2C) has a resolution 13Å (Fig 2D); it possesses a larger number of subunits per turn (~ 9.55), with the same pitch, as the wild type AltMV virion (~ 35.7 Å). The central channel of VLP particle is somewhat larger with the diameter ~30Å and its walls have pronounced gaps (arrows on Fig 2C, below). This may account for lacking the density, attributed to the viral RNA (arrows in Fig 1C and 1D). To demonstrate the difference between AltMV VLP and virion particles, we generated the difference map by subtracting the VLP density from the virion density (Fig 3). The difference density (in red) clearly marks the tracks of the viral RNA inside the wild type virion (Fig 3A). Comparison of the sagittal sections of the AltMV VLP and virion (Fig 1D and Fig 2C, below) revealed that the CP makes the stable VLP in the absence of viral RNA. This is consistent with the fact that in known high resolution structures of Potexviruses [11–13] major intersubunit contacts include both N- and C-termini which produce the interconnected net. Here we demonstrate that RNA contacts are not required to build the stable virus-like particle.

According to our recent data, native AltMV-MU virions and VLPs are antigenically related, but not identical [15]. This indirectly indicates that the overall structure of AltMV virions and VLPs can differ despite their morphological similarity. To demonstrate this, a ‘trypsin test’ developed earlier in our laboratory [30] was used to compare AltMV virions and VLPs. This method earlier allowed us to determine the oligomeric state of potato virus X (PVX) CP and to distinguish whether its CP is present in the solution in monomeric (dimeric) form or is oligomerized to form the viral capsid [30].

Here, we demonstrated that upon trypsinolysis the CPs of AltMV virions remained intact (Fig 2E, lane 2) similarly to the control sample (without trypsin) (Fig 2E, lane 1). At the same conditions, the purified CP oligomerized into VLPs underwent partial hydrolysis (Fig 2E, lane 4) as compared to control (Fig 2E, lane 3). It is known that AltMV-MU CP possesses 11 potential sites of trypsin hydrolysis with a probability of 100%, one site with a probability of 75% and one with a probability of 40% [10]. However, in the native virion neither of them is exposed to the enzyme digestion, in contrast to VLPs where part of hydrolysis sites are available for trypsinolisis. The morphology of untreated and trypsin-treated AltMV virions and VLPs was compared by electron microscopy and was shown to be similar (S3B and S3D Fig). Thus, we can hypothesize that, despite the similarity in the overall morphology when studied at low magnification, the folding and intersubunit interactions of AltMV CP differ in the presence and absence of RNA. This is consistent with previous data for the antigenic properties of AltMV virion versus AltMV VLP [15], and with the recent data for PapMV trypsinization [37].

The VLPs are stable under the same conditions as the native AltMV virions. VLPs do not change their morphology and size during incubation in distilled water (S4A Fig), in 0.15 M NaCl (S4B Fig), and 0.01 M Tris-HCl, 0.15 M NaCl, pH 7.5 (S4C Fig). The absence of RNA in the particles and, therefore, the absence of RNA-protein interactions did not affect the stability of the protein helix of the AltMV VLPs under the selected conditions. Particularly worth mentioning is that AltMV VLPs also remained stable after 1 hour incubation in mouse serum (S4D Fig).

Thereby, AltMV VLPs have marked advantages in comparison with VLPs obtained from the PapMV CP. The PapMV CP assembly into full-length VLPs only at pH 4.0, whereas at pH 8.0 they only form small helical aggregates (sedimentation coefficients ~13-33S). Adding of 0.2 M NaCl at pH 4.0 results in the assembly of much shorter VLPs [14,38]. In contrast, AltMV CP readily polymerizes *in vitro* into RNA-free VLPs under various conditions, including physiological pH 7.5 and in the presence of NaCl, as well as in the serum of laboratory animals (S4 Fig).

## Conclusions

Undoubted interest in plant viruses, virus-like particles (VLPs) and the possibility of their use in the development of new biotechnologies (in medicine, veterinary, microelectronics, etc.) is constantly growing. Obtaining of VLPs under various conditions, and study of the structure, properties and stability of virions and VLPs is a direction of research of great significance. The economically expedient use of viruses in biotechnology is possible only if they are produced in large quantities. Therefore, in this work the procedure for the Alternanthera mosaic virus (AltMV) isolation was modified. As a result, the yield of AltMV was significantly increased (up to 57.3 mg of virus/100 g of plant biomass).

Here, for the first time, the 3D structures of AltMV virions and its VLPs were obtained by single particle EM at ~13Å resolution. The comparison of the reconstructions revealed that AltMV CPs might have a different fold in the presence and absence of viral RNA. This has been confirmed by a trypsin hydrolysis. Thus, for the first time the structure of morphologically similar virions and virus-like particles based on the coat protein of a helical filamentous plant virus is shown to be different.

AltMV virions are demonstrated to be stable in various conditions, including the serum of laboratory animals. AltMV coat protein (CP) is shown to be able to form *in vitro* stable extended polymers–RNA-free VLPs–under different conditions; VLPs are identical in morphology, but differ in structure to the AltMV virions. The principal difference between VLPs of AltMV and VLPs of the related papaya mosaic virus is the high stability of AltMV VLPs in a wider range of conditions, including physiological, and the absence of nucleic acid in their composition.

## Supporting information

S1 Fig Single particle EM analysis of AltMV virions and VLPs structure. **A.** AltMV virions, **B.** AltMV VLPs. Top row–segments, cut from raw image; below–corresponding class averages. Right–width determination of the helical particle.
(TIF)

S2 Fig Analysis of AltMV virions in different conditions. **A.** in distilled water, **B.** in 0.15 M NaCl, **C.** in 0.01 M Tris-HCl, 0.15 M NaCl, pH 7.5, **D.** in mouse serum. Incubation for 1 hour. TEM, staining with 2% uranyl acetate.
(TIF)

S3 Fig Analysis of AltMV virions and VLPs morphology after trypsin treatment. **A.** AltMV virions, **B.** AltMV virions treated by trypsin, **C.** AltMV VLPs, **D.** AltMV VLPs treated by trypsin. Incubation with the enzyme for 1 hour. TEM, staining with 2% uranyl acetate.
(TIF)

S4 Fig Analysis of AltMV VLPs in different conditions. **A.** in distilled water, **B.** in 0.15 M NaCl, **C.** in 0.01 M Tris-HCl, 0.15 M NaCl, pH 7.5, **D.** in mouse serum. Incubation for 1 hour. TEM, staining with 2% uranyl acetate.
(TIF)

## Abbreviations

AltMV: Alternanthera mosaic virus
CP: coat protein
EM: electron microscopy
FSC: Fourier shell correlation
PapMV: papaya mosaic virus
TEM: transmission electron microscopy
VLP: virus-like particle
