# Supplementary figures and images for: Structure and properties of virions and virus-like particles derived from the coat protein of Alternanthera mosaic virus
Source: PLoS One. 2017 Aug 24;12(8):e0183824. doi: 10.1371/journal.pone.0183824 (PMC5570366; doi:10.1371/journal.pone.0183824)

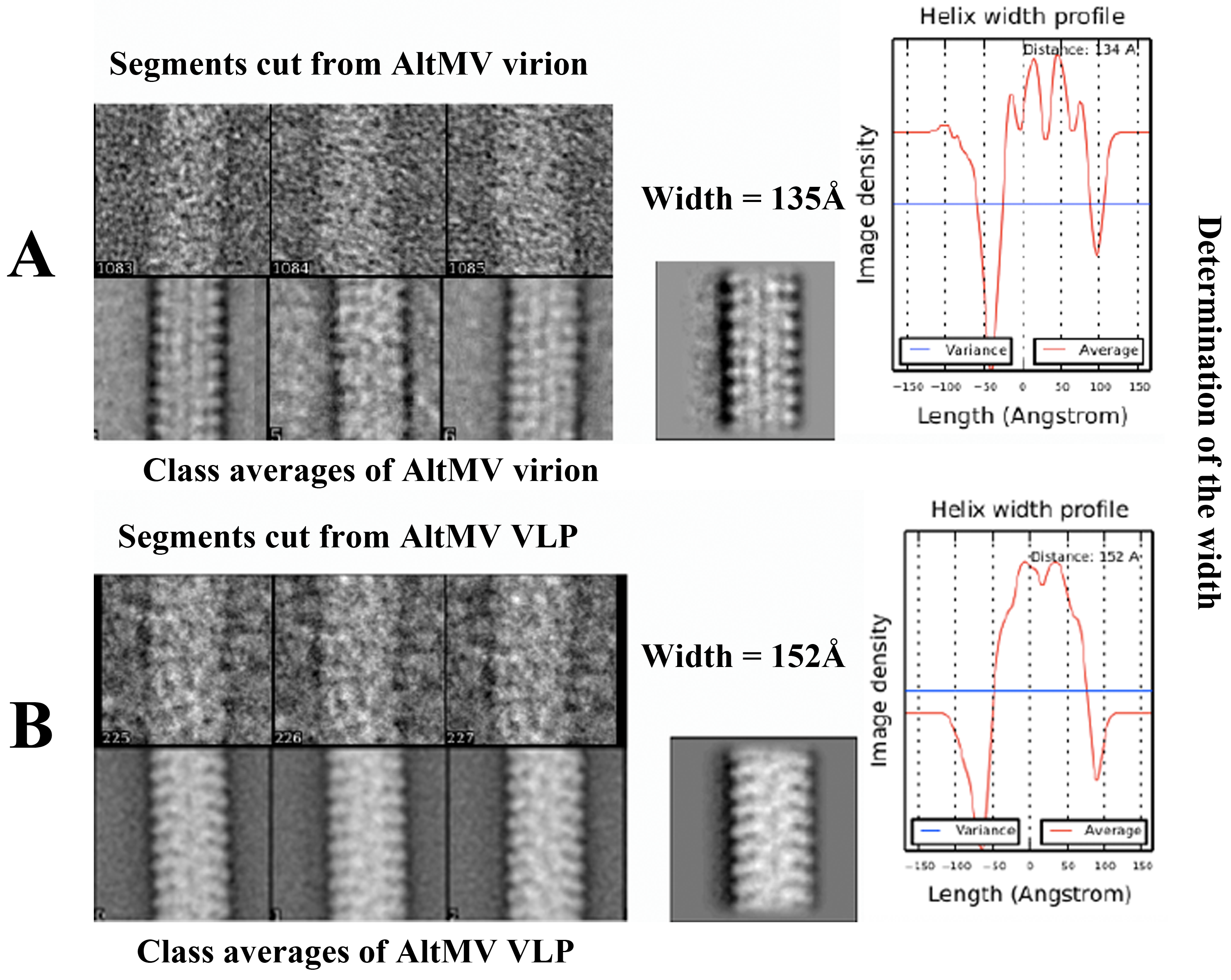

Supplement: S1 Fig — A. AltMV virions, B. AltMV VLPs. Top row–segments, cut from raw image; below–corresponding class averages. Right–width determination of the helical particle. (TIF) [file pone.0183824.s001.tif]

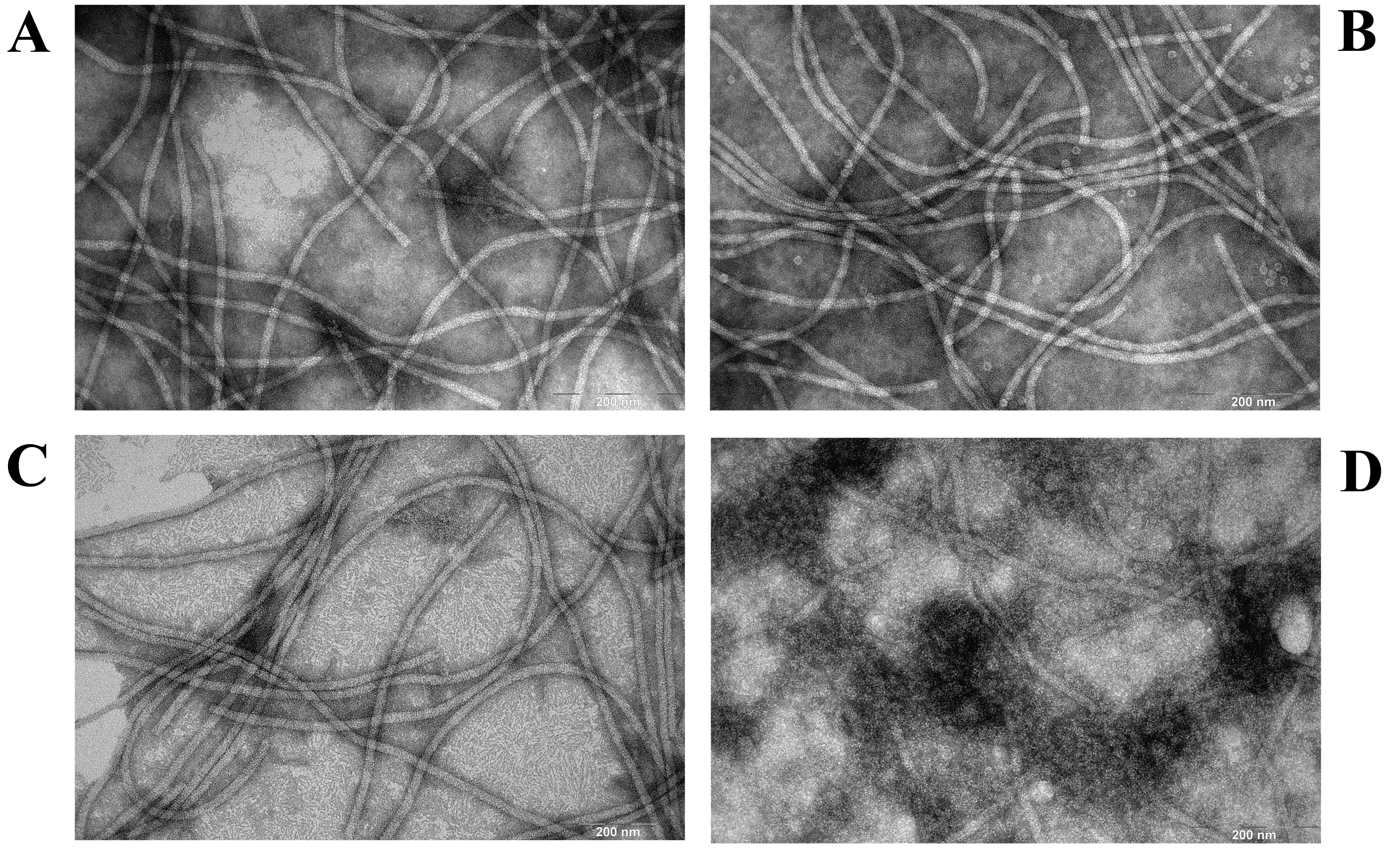

Supplement: S2 Fig — A. in distilled water, B. in 0.15 M NaCl, C. in 0.01 M Tris-HCl, 0.15 M NaCl, pH 7.5, D. in mouse serum. Incubation for 1 hour. TEM, staining with 2% uranyl acetate. (TIF) [file pone.0183824.s002.TIF]

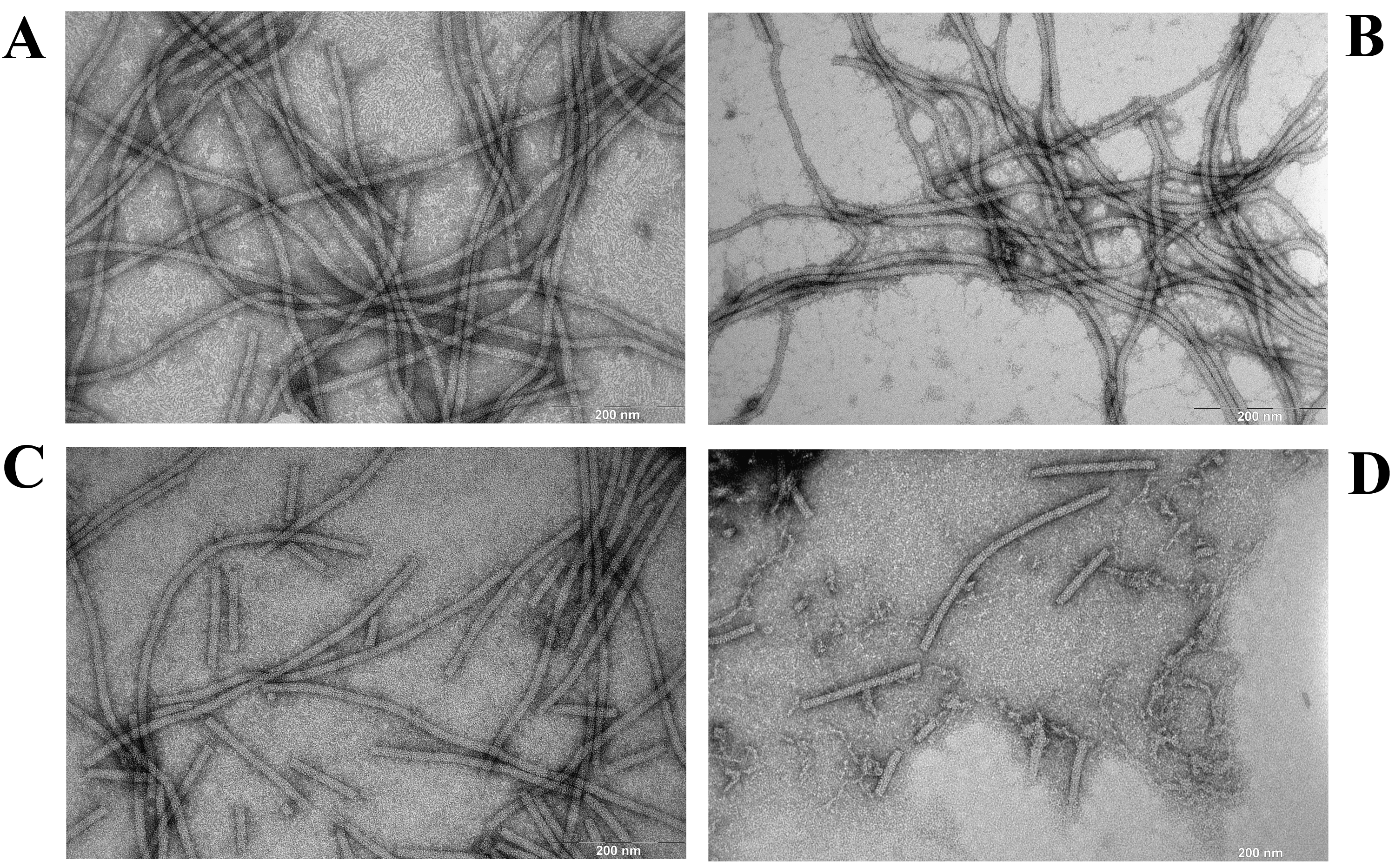

Supplement: S3 Fig — A. AltMV virions, B. AltMV virions treated by trypsin, C. AltMV VLPs, D. AltMV VLPs treated by trypsin. Incubation with the enzyme for 1 hour. TEM, staining with 2% uranyl acetate. (TIF) [file pone.0183824.s003.TIF]

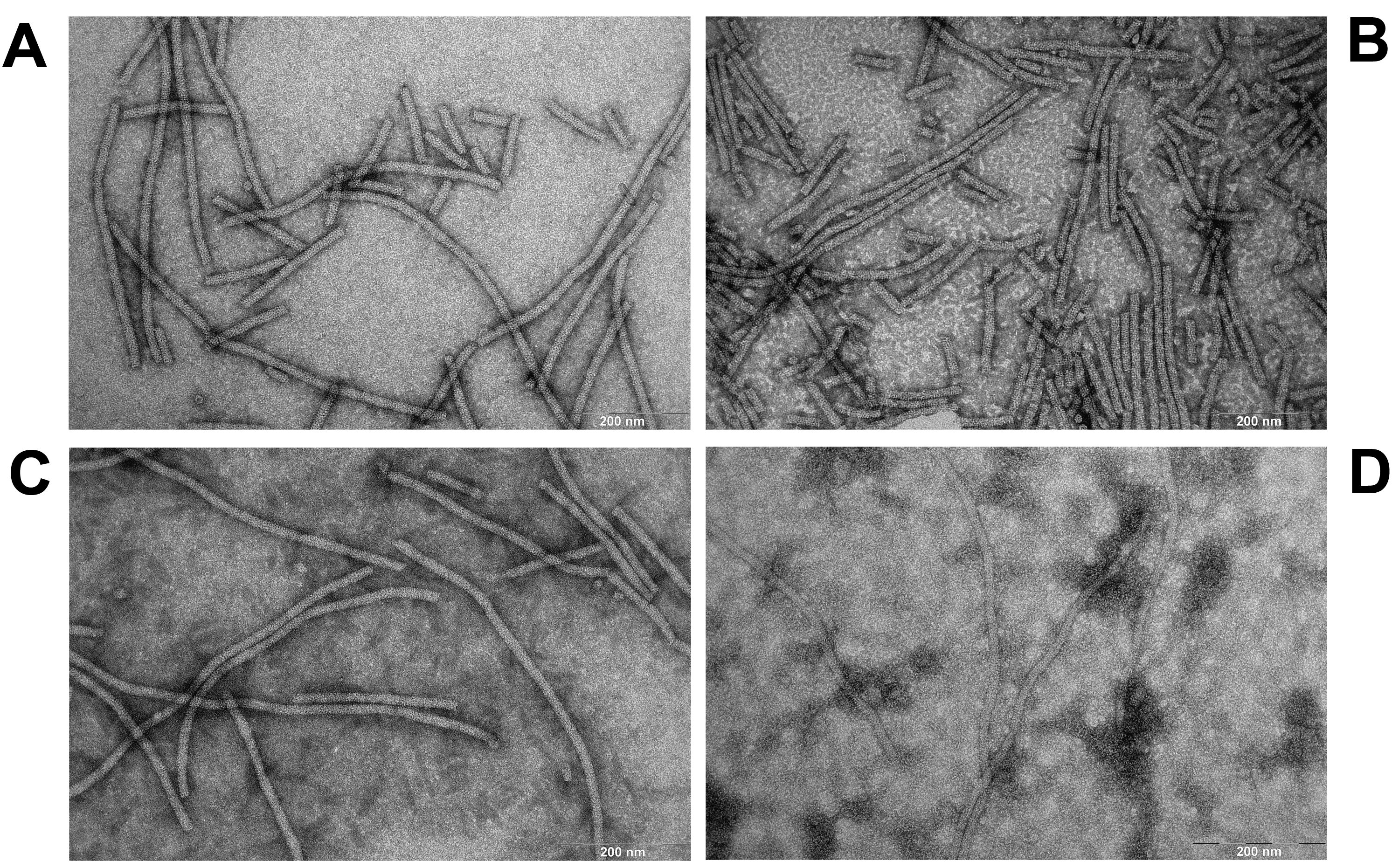

Supplement: S4 Fig — A. in distilled water, B. in 0.15 M NaCl, C. in 0.01 M Tris-HCl, 0.15 M NaCl, pH 7.5, D. in mouse serum. Incubation for 1 hour. TEM, staining with 2% uranyl acetate. (TIF) [file pone.0183824.s004.TIF]
